# Supplementary material for: Predicting cardiopulmonary exercise testing outcomes in congenital heart disease through multimodal data integration and geometric learning
Source: Sci Rep. 2026 Feb 19;16:9910. doi: 10.1038/s41598-026-38687-1 (PMC13018615; doi:10.1038/s41598-026-38687-1)
Supplement: Supplementary file 1 — Supplementary Information. [file 41598_2026_38687_MOESM1_ESM.pdf]

Manuscript Title: Predicting Cardiopulmonary Exercise Testing Outcomes in Congenital Heart Disease Through Multimodal Data Integration and Geometric Learning
Manuscript ID: ad2fd1d0-ab68-4b17-bd6e-934375b5586a v3.0

Supplementary Figure 1

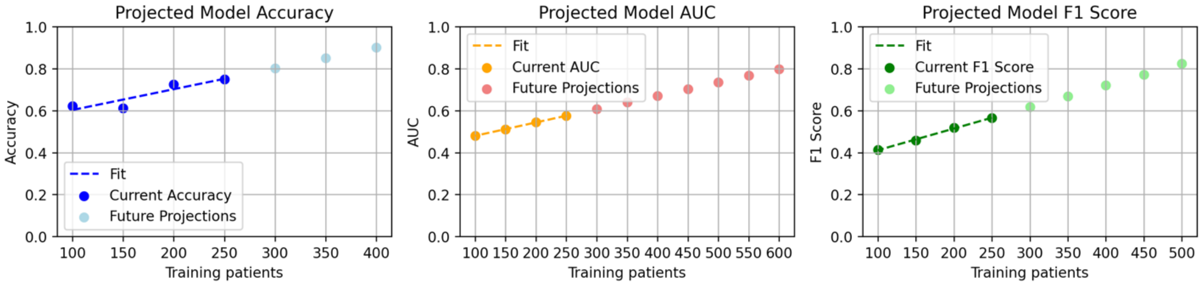

Figure S1: Current and projected metrics for clinically relevant outcomes: the effect of increasing training patient numbers on accuracy, AUC, and F1 score.

Figure S1 presents the projected model scores for VE/VCO_2, providing accuracy, AUC and the F1 score, across a range of sample sizes up to 400 patients. This analysis reveals that with 300 patient samples, accuracy reaches at 0.81, AUC reaches 0.61, and F1 score reaches at 0.62. As the dataset expands to 400 patient samples, accuracy improves to 0.89, AUC rises to 0.68, and F1 score reaches 0.73. This trend underscores the advantage of incorporating more patient data, as it enables the model to learn more complex patterns and enhance prediction performance. These findings underscore the importance of continuous data collection and model refinement to optimise predictive accuracy in healthcare applications. We acknowledge that the linear relationship is indeed an approximation of potential performance trends. However, given our extremely small sample size, a linear approximation is not unreasonable and demonstrates a good fit to the observed data points (r2 score for accuracy is 0.869, for AUC is 0.968, and for F1 score is 0.924) giving us a signal of potential value. Our intention was to provide a preliminary visualization of potential performance trends, recognizing the inherent limitations of such a representation.

Supplementary Figure 2

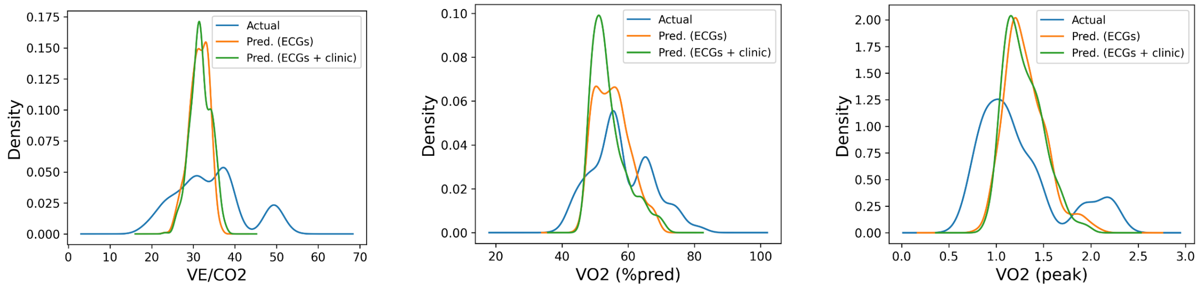

(a) VE/VCO_2 (b) VO_2 (%pred) (b) VO_2 (peak)

Figure S2: Kernel Density Estimations (KDEs) of the SVM model.

Figure S2 illustrates the distribution of the actual and predicted values, indicating that the predictions of the model with multi-modal data are much closer to the distribution of actual exercise values. These plots also demonstrate a positive alignment with the improved results.

Supplementary Figure 3

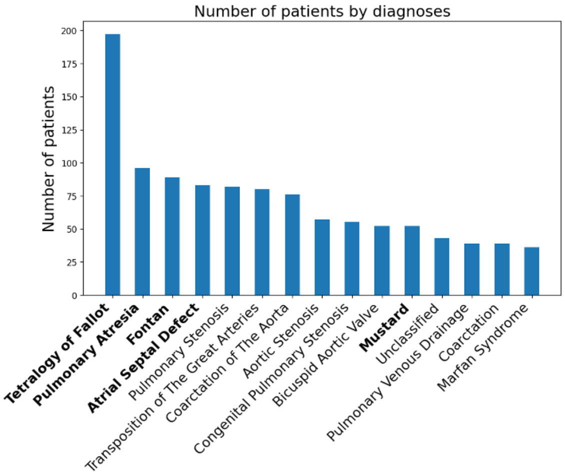

Figure S3: Top 15 anatomic diagnoses for 1409 patients.

Figure S3 presents a summary of the top 15 anatomic diagnoses among 1409 patients in the study approved by the Institutional Governance Division of the NHS Golden Jubilee National Hospital. Demographic information including age, gender, anatomic diagnoses, and prior surgical intervention were extracted from clinical letters as previously described in [1]. The most common condition was tetralogy of Fallot in 197 patients, followed by pulmonary atresia in 96 patients.

[1] Verma, S., Alkan, M., Deligianni, F., Anagnostopoulos, C., Diller, G., Walker, L., Johnston, F., Danton, M., Walker, H., Swan, L. & Others Development of a Semiautomated Database for Patients with Adult Congenital Heart Disease. Canadian Journal of Cardiology. 38, 1634-1640 (2022)

Supplementary Figure 4

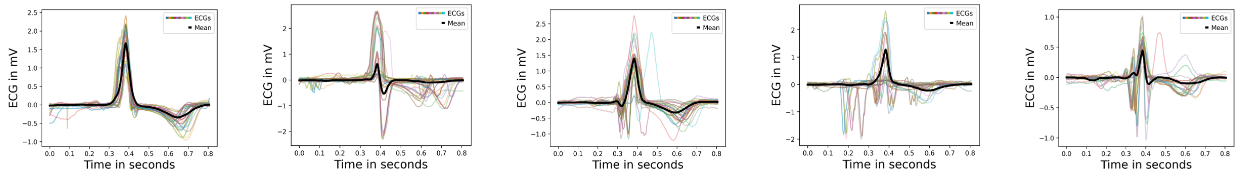

(a) Mustard, Lead v1 (b) Fontan, Lead v1 (c) ToF, Lead v1 (d) PA, Lead v1 (e) ASD, Lead v1

Figure S4: Aligned ECGs on lead v1 of patients with the specified conditions (mean signal outlined in bold).

Figure S4 presents the average signal of the aligned ECGs for the specified conditions and its characteristic of the underline abnormality. To prepare the ECG data for machine learning analysis, we standardised the signals by aligning the R peaks across all heartbeats and leads. This preprocessing step ensured digital synchronization of QRS complexes, creating a uniform temporal reference point across all patient recordings. The average length of non-aligned ECGs is 2.5 seconds and R peak aligned ECGs is around 1 second, and they are sampled at a rate of 500 samples per second. The ECG recordings were sampled at 500 Hz, with unaligned segments spanning 2.5 seconds and R-peak aligned segments spanning approximately 1 second.

Supplementary Table 1

Table S1: Number of patients excluded from the study due to the specified criteria.

Condition	Initial Patients	Excluded Patients	Study Patients
ToF	197 patients	24 patients	173 patients
PA	96 patients	23 patients	73 patients
Fontan	89 patients	23 patients	66 patients
ASD	83 patients	6 patients	77 patients
Mustard	52 patients	5 patients	47 patients

A total of 81 patients as outlined in Table S1 were excluded due to the specified criteria of having ECGs showing atrial flutter, atrial fibrillation, or atrioventricular paced rhythms. We acknowledge that these conditions represent a significant patient group often associated with poorer prognoses, and this strict exclusion process does indeed narrow the patient population under study. We have added a discussion to address this concern, emphasising the potential implications of excluding these patients and suggesting that future research could explore the outcomes in patients with arrhythmias to provide a more comprehensive understanding of the overall patient population.
